# Quantitative imaging of mammalian transcriptional dynamics: from single cells to whole embryos

**DOI:** 10.1186/s12915-016-0331-9

**Published:** 2016-12-23

**Authors:** Ziqing W. Zhao, Melanie D. White, Stephanie Bissiere, Valeria Levi, Nicolas Plachta

**Affiliations:** 1 Institute of Molecular and Cell Biology, Agency for Science, Technology and Research (A*STAR), 61 Biopolis Drive, Singapore, 138673 Singapore; 2 Facultad de Ciencias Exactas y Naturales, Universidad de Buenos Aires, Conicet, Buenos Aires C1428EHA Argentina

## Abstract

Probing dynamic processes occurring within the cell nucleus at the quantitative level has long been a challenge in mammalian biology. Advances in bio-imaging techniques over the past decade have enabled us to directly visualize nuclear processes in situ with unprecedented spatial and temporal resolution and single-molecule sensitivity. Here, using transcription as our primary focus, we survey recent imaging studies that specifically emphasize the quantitative understanding of nuclear dynamics in both time and space. These analyses not only inform on previously hidden physical parameters and mechanistic details, but also reveal a hierarchical organizational landscape for coordinating a wide range of transcriptional processes shared by mammalian systems of varying complexity, from single cells to whole embryos.

## The “space-time” of the cell nucleus and techniques for its imaging

The nucleus is an organelle of central importance to the eukaryotic cell, in which the information encoded in the cell’s genome is stored, organized, expressed, duplicated, and maintained. Each of these processes is highly regulated, often in an interconnected fashion. While we now have a relatively thorough understanding of the molecular machineries and mechanisms driving these processes, our knowledge of how they are organized spatially inside the nucleus remains inadequate. Such a question is particularly pertinent in light of the fact that all of these processes co-exist in the extremely crowded nuclear space, thus suggesting that some degree of functional compartmentalization is essential [1, 2]. Moreover, even in cases where the “geography” of a nuclear process is known (either in Cartesian space or sequence space), its temporal dynamics often remain poorly characterized. Since many nuclear proteins move rapidly and interact with various nuclear compartments [3], these dynamic events, which can be likened to the “historical” details of mammalian nuclear biology, provide critical insights into how these molecules search for and reach their specific targets to carry out their respective functions, all within this dense and yet ordered nuclear “space-time”. These inadequacies in understanding call for novel ways of probing the nucleus by visualizing these structures and processes in situ in single cells, with high spatial and temporal resolutions and, ideally, single-molecule sensitivity.

**Fig. 1.**
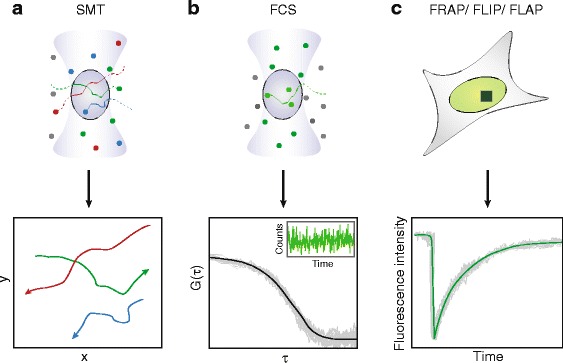
Optical techniques useful for imaging the mammalian cell nucleus in space and time. **a** Single-molecule tracking (*SMT*) using epi-illumination, in which fluorescently labeled molecules within the laser focus (*purple oval*) are excited and their movements followed over time; a few representative single-molecule trajectories are depicted. **b** Fluorescence correlation spectroscopy (*FCS*), which analyzes the fluctuations in fluorescence intensity as molecules move in and out of the laser focus to obtain quantitative information on their dynamics; a representative intensity fluctuation trace (*inset*) and the autocorrelation function curve calculated from the trace are shown. **c** Photobleaching-based imaging techniques, depicting a small region of the nucleus (*green*) that has been selectively photobleached (*dark green box*); a typical FRAP curve is shown here (*red arrowhead* denotes photobleaching)

Among the imaging techniques currently available, the most widely used as well as the most direct method is perhaps single-molecule tracking (SMT), which relies on the ability to detect the signal of individual biomolecules labeled with either fluorescent proteins or organic dyes [4, 5]. While those molecules undergoing rapid movement would contribute to a diffuse fluorescence background, those that are immobile or bound give rise to distinguishable signals above the background, thus allowing their positions to be localized and their dynamics tracked over a period of time (Fig. 1a). However, the relative thickness of the mammalian cell nucleus, its high auto-fluorescence background, and the fact that many of the key molecular species are present at high copy numbers [6] make single-molecule detection in the nucleus challenging. This problem is particularly pronounced when using wide-field epi-fluorescence microscopes, which excite all molecules along the illumination path, leading to higher background that could easily overwhelm the signals of individual molecules. To circumvent this difficulty, various schemes have been implemented to reduce the excitation volume beyond that afforded by epi-illumination and enhance sensitivity. In addition to earlier solutions such as total internal reflection fluorescence (TIRF) and highly inclined and laminated optical sheet (HILO) [7] microscopies, more recent efforts leverage the superior optical sectioning capability of light-sheet microscopy (also termed selective plane illumination microscopy (SPIM)) and have successfully achieved single-molecule detection inside the cell nucleus [8–10] as well as super-resolution imaging capable of resolving nuclear structures beyond the diffraction limit [8, 11–13]. While fluorescent proteins (FPs) such as GFP are still a common choice for labeling proteins of interest, recently developed tags such as SNAP [14], CLIP [15], and Halo [16] allow organic dyes, which are brighter and more photostable than FPs, to be used as fluorescent labels in live cells. In addition to following protein molecules, labeling methods such as MS2 [17], PP7 [18], or RNA-targeting Cas9 [19] have also enabled live-cell detection of individual RNAs, while other techniques such as single-molecule fluorescence in situ hybridization (smFISH) [20], although incapable of capturing dynamic information in live cells, can nonetheless probe dynamic phenomena by providing high-resolution snapshots of RNA transcripts at defined time points.

Another powerful approach is fluorescence correlation spectroscopy (FCS), which consists of a compendium of related techniques [21–27] based on the analysis of intensity fluctuations produced when fluorescent molecules move in and out of a small observation volume (Fig. 1b). Instead of tracking individual molecules, these fluctuation traces are subjected to autocorrelation analysis, a mathematical algorithm capable of detecting patterns in temporal signals, allowing quantitative information on the dynamics of the molecules to be extracted. The temporal window of the fluctuations depends on the photophysics of the fluorescent molecules as well as their mobility, and can thus span timescales from microseconds to seconds. As such, FCS is capable of probing a wide range of dynamic processes in living systems, including diffusion, transport, and binding interactions [28, 29], and the analysis can be complemented with Monte Carlo simulations to uncover the dynamics of even more complex processes [30, 31]. FCS can be combined with photoactivation (paFCS) to fine-tune the number of fluorescent molecules detected by selectively activating only a desired fraction of the molecules [32], thereby enhancing the signal-to-noise ratio and making it suitable for systems with high fluorescence background. Integrating SPIM with FCS has also enabled massively parallelized (instead of point-by-point) data acquisition schemes [33, 34], leading to higher imaging speed and spatially resolved mobility and binding maps. Other related fluctuation-based techniques, such as photon counting histogram (PCH) [35] and number and brightness (N&B) analysis [36], are particularly suited for probing the concentration and oligomerization state of biomolecules.

A third group of imaging approaches consists of photobleaching-based techniques such as fluorescence recovery after photobleaching (FRAP), fluorescence loss in photobleaching (FLIP), and fluorescence localization after photobleaching (FLAP) [37–39]. While all three involve photobleaching fluorescent molecules in a localized region of the cell using intense laser illumination, FRAP monitors the replenishing of these molecules into the region after a single photobleaching (Fig. 1c), FLIP tracks how the loss of fluorescence propagates through the cell upon repeated photobleaching, and in FLAP two different colocalizing fluorophores are present in the region and photobleaching is performed on only one of them. Owing to the complexity of the processes at work, intense efforts have also been made regarding the rigorous analysis of these datasets using various versions of the reaction-diffusion model in order to extract dynamic information more accurately [40–43]. In addition, related techniques such as pixel-wise photobleaching profile evolution analysis (3PEA) can extend the temporal resolution of FRAP to the millisecond regime, making it suitable for monitoring fast and transient events in the cell [44].

Each of these techniques has its respective pros and cons. SMT has the advantage of allowing dynamics to be visualized directly without the need for additional calibrations and corrections commonly associated with the other techniques [45]; however, the relatively short trajectories that can be captured (especially when using fluorescent proteins) could limit its scope and utility. The photobleaching-based techniques, on the other hand, are capable of probing dynamics at longer timescales. However, given that such probing involves a population (or ensemble) of molecules, these techniques are prone to masking the intrinsic heterogeneities among individual molecules; after all, many of the nuclear processes (such as transcription or replication) involve only one or two DNA molecules and a small number of enzyme or regulatory molecules per event [46]. FCS has perhaps the widest span in temporal coverage and uses less laser power, thus reducing potential photodamage to the sample compared to photobleaching-based methods. On the other hand, the requirement of nanomolar concentrations of fluorescent probes inevitably introduces some perturbation to the system under study. Lastly, in the spatial realm, the fact that the positions of individual molecules could be pinpointed with nanometer precision potentially allows SMT and smFISH to achieve resolutions beyond the diffraction limit [47–49], giving it another competitive edge over the other techniques, which remain diffraction-limited.

In this review, we survey some of the key recent studies on imaging mammalian nuclear dynamics in both time and space using these emerging approaches as well as some of the more conventional techniques (such as confocal microscopy). To that end, we focus on processes related to transcription, not only because of the tremendous progress made in recent years in understanding its quantitative dynamics in living systems [50–54], but also for the fact that its organization encompasses a wide variety of spatio-temporal modes commonly employed to regulate many other nuclear processes.

## Temporal organization of transcriptional dynamics

Eukaryotic transcription is regulated first and foremost through the binding and unbinding of a variety of transcription factors (TFs), as well as their interactions with components of the transcriptional machinery, chief among them RNA polymerases, during transcription initiation. Mammalian TFs have long been known to interact with DNA in a highly dynamic manner [55]. Concurrent with the rapid progress in mapping the genomic binding sites of a large number of these factors using chromatin immunoprecipitation (ChIP) and high-throughput sequencing technologies [56], quantitative imaging using SMT, FCS, or photobleaching-based approaches have recently shed light on how such binding events occur in time.

A variety of TF dynamics have so far been investigated, including their interactions with genomic DNA, co-activators, and heterodimeric binding partners. Specifically, distinct modes of TF–DNA binding have been resolved, and diffusion constants, on/off rates, DNA residence times, and bound and free fractions have been measured for a diverse range of TFs, including, for example, the glucocorticoid receptor (GR) [9, 40, 42, 57–62], estrogen receptor (ER) [9, 61, 63], Sox2 and Oct4 [32, 64–66], p53 [42, 59, 67, 68], c-Myc [69], positive transcription elongation factor (P-TEFb) [69], cAMP response element-binding protein (CREB) [70], signal transducer and activator of transcription 1 (STAT1) [71], retinoic acid receptor (RAR) [72], vitellogenin binding protein (VBP) [73], and heat shock factor 1 (HSF1) [74]. For most TFs, specific and nonspecific binding exhibit a clear separation of timescale, with the residence time for the shorter-lived nonspecific binding falling in the range of tens to hundreds of milliseconds, and that for the longer-lived specific binding in the range of hundreds of milliseconds to a few seconds [9, 40, 42, 59–61, 64, 66, 67, 70, 71]. Of special note is the fact that, after years of improvement in analysis procedures, consistent values of residence times and bound fractions have been reached for both GR and p53 using SMT, FCS, and FRAP [60, 67, 75], suggesting that we are at last in possession of a set of self-consistent and complementary techniques for probing the dynamics of TFs and other nuclear proteins.

**Fig. 2.**
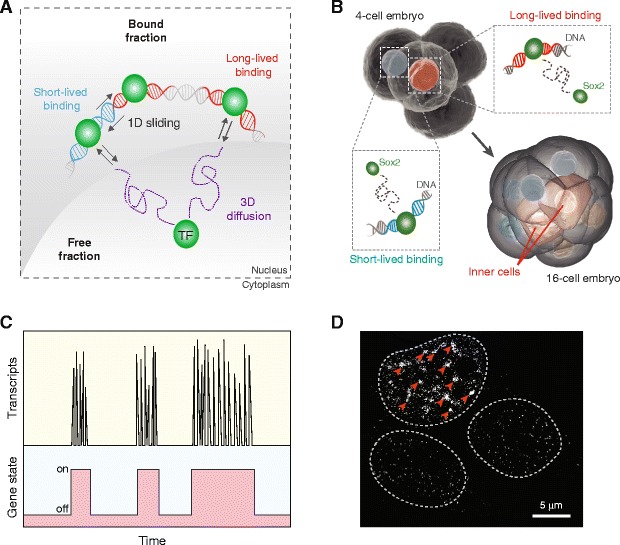
Temporal modes of organizing mammalian transcriptional dynamics. **a** Modulating transcription through TF binding and target search, in which a TF molecule undergoes “facilitated diffusion” by partitioning its movement between free 3D diffusion (*purple*) and transient 1D sliding along the DNA (*blue*) until the specific target sites are located and to which the TF stays bound for a long time (*red*). **b** An example of the physiological consequence of TF binding dynamics, in which the long-lived bound fraction of Sox2 in a blastomere of a four-cell embryo predicts the bias with which this blastomere will contribute to the inner mass of the embryo subsequently. **c** Modulating transcription through pulsatile production or “bursts” of mRNAs, as a consequence of stochastic switching of the gene between the “on” and ”off” states. **d** Widely different Nanog expression among a population of mouse ES cells as revealed by smFISH. In contrast to the sparse Nanog molecules present in the two lower cells, multiple spots where bursts of Nanog transcription took place (indicated by *red arrowheads*) are discernible in the top cell. *Dotted lines* delineate nuclear boundaries. Adapted from [66] (**a, b**) with modifications

Using the dynamic parameters uncovered, models for how mammalian TFs search for their specific targets within the nuclear space have been constructed. In consensus with the target search mechanism in bacteria [76], mammalian TFs also scan the genome by undergoing transient 1D sliding along nonspecific DNA sites interspersed with 3D diffusion steps between different regions of the genome, before reaching their specific targets (Fig. 2a). Such “facilitated diffusion” [77] enhances the efficiency of the search process and is exhibited by TFs such as Sox2 [64, 65] and STAT1 [71]. Similar mechanisms have also been found for non-endogenous DNA-binding proteins such as Tet repressor [78] and CRISPR-associated Cas9 [79], suggesting a conserved approach to target search in the mammalian nucleus shared by a wide range of genome interactors, both eukaryotic and prokaryotic in origin.

While the above approaches have been successful mainly in cultured cell systems, much less work has been done in more in vivo contexts. Recently, the nuclear dynamics of two TFs critical for early mammalian development, Oct4 and Sox2, were probed inside intact mouse embryos [32, 66, 80]. Similar to the case in cultured cells, in the embryo these TFs display both fast Brownian diffusion and slower anomalous diffusion [32], with the latter arising from both short- and long-lived DNA interactions. Remarkably, the longer-lived bound fraction of Sox2 in each blastomere of the four-cell embryo directly correlates with the number of pluripotent progeny that cell will later contribute to the inner mass of the embryo (Fig. 2b) [66]. These findings demonstrate the dynamic repartitioning of TFs between distinct DNA sites in vivo, and show that quantitative changes in TF–DNA interactions could have physiological consequences directing embryonic cell fate at a very early stage of development.

Aside from TF binding, the process of transcription itself is also temporally heterogeneous, manifested primarily in the form of “transcriptional bursts” (the production of nascent mRNAs in a pulsatile fashion), in which each pulse consists of a burst of transcript molecules and is separated from the next by a period of inactivity (Fig. 2c). Such Poisson-like bursts often occur on a timescale ranging from minutes to hours, and have been observed in a variety of mammalian systems [81–89]. In fact, “bursty” transcription has been found to be the predominant form of expression for 8000 different loci in the human genome [88]. More recently, transcriptional bursts have also been detected in intact mammalian tissues such as liver [90], suggesting that this mode of temporal organization might indeed be at work in vivo.

The dynamics of transcriptional bursts are often gene-specific, and can be regulated at many levels. Single-cell luminescence measurements on a diverse range of mammalian genes have revealed distinct temporal patterns of mRNA synthesis, manifested in both the on/off rates of transcription and the mean numbers of mRNA molecules produced per burst (or burst size) [87]. In the human genome, weaker expression loci have been found to primarily modulate their burst frequency, while stronger expression loci modulate their burst size [88]. In other cases such as steroid receptor-mediated transcription, gene activation through ligand-binding alters neither the burst size nor the duration of each burst, but only the duration of the refractory periods between bursts. Such “frequency modulation” at the single-cell level could give rise to dose-dependent responses in a population of cells [82]. Mechanistically, transcriptional bursts are produced as a consequence of the stochastic switching of the promoter between transcriptionally active (“on”) and inactive (”off”) states [91], a process that is contingent upon TF binding/unbinding and modulated by a variety of other factors including promoter architecture [87], different physiological stimuli (such as serum, growth factors, and so on) [83], and strength of the TF’s transactivation domain [85].

The physiological consequences of transcriptional bursts are profound. They are known to cause cell-to-cell variability in gene expression (“noise”), which could in turn generate opportunities for an otherwise isogenic population of cells to explore different phenotypes or lineage fates [91, 92]. For example, in mouse embryonic stem (ES) cells, pulsatile transcription of Nanog has been shown to result in widespread stochastic fluctuations inherent to the pluripotent state (Fig. 2d). More importantly, cells with low Nanog levels are more prone to enter differentiation due to the expression of lineage marker genes [93]. Such fate-determining capability, together with its diverse forms of modulation, makes pulsatile dynamics a pervasive temporal mode for organizing transcription.

## Spatial organization of transcriptional dynamics

**Fig. 3.**
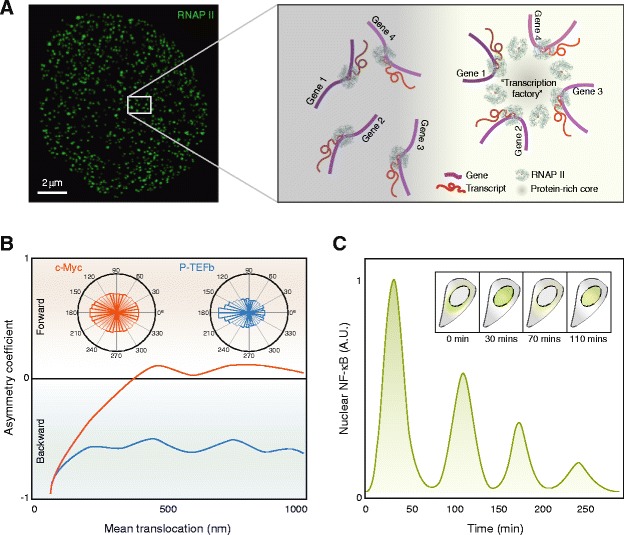
Spatial modes of organizing mammalian transcriptional dynamics. **a** A super-resolution map of RNAP II distribution inside a mammalian cell nucleus. *Inset* shows a zoom-in area illustrating the co-existence of both isolated RNAP II molecules (*left*) as well as transient clusters (“transcription factories”, *right*) that coordinate the expression of spatially disparate genes. **b** When the angles between successive translocation steps of TF molecules during target search are measured by SMT, P-TEFb (*blue*) exhibits substantial asymmetry (evidenced by the strong bias toward 180° in the angular distribution and the negative asymmetry coefficient), indicating a propensity to “back-stepping” due to spatial constraints of the search process. In contrast, c-Myc (*orange*) explores the nuclear space more or less unhindered with no preferred directionality (evidenced by the near-zero asymmetry coefficient). **c** Modulating transcription through oscillatory nucleo-cytoplasmic translocation of NF-κB; single-cell snapshots of nuclear NF-κB level at representative time points are depicted in *insets*. Adapted from [12] (**a**), [69] (**b**), and [101] (**c**) with modifications

Similar to their temporal heterogeneity, transcriptional processes are also unevenly distributed in the nuclear space, and such spatial organization plays an important role in their regulation. A prime example is transcription mediated by RNA polymerase II (RNAP II). Based on earlier observations that nascent mRNA transcripts tend to localize to discrete foci inside the nucleus, the hypothesis of “transcription factories” (Fig. 3a), nuclear sub-structures consisting of multiple clustered RNAP II molecules that carry out the coordinated transcription of multiple genes, was conceived [94, 95]. Recent super-resolution studies in live cells have observed transient dynamic assembly of RNAP II into short-lived clusters, which correlate with nascent mRNA production [96, 97]. These clusters, with a lifetime on the order of seconds, have been found to assemble and disassemble “on demand” in an asynchronous fashion [97, 98]. Such findings are in line with a similar study performed in fixed cells using a global molecular counting approach, which found that a small fraction of all RNAP II molecules in the nucleus colocalize with each other at any given moment, while the rest exist in an unclustered form [12]. These two perspectives represent two complementary manifestations of the “ergodic principle”, which suggests the equivalence between time-averaging and ensemble-averaging, as applied in the context of intranuclear RNAP II clustering. Moreover, live-cell imaging of cyclin-dependent kinases associated with different phosphorylation forms of RNAP II further suggest that the initiation and elongation of mRNA transcripts may also take place in mutually exclusive nuclear compartments, and that elongating RNAP II, upon phosphorylation at the Ser2 position, moves out of those “factories” where Ser5-phosphorylated RNAP II carries out transcriptional initiation [99].

RNAP II is by no means the only molecule whose intranuclear localization is regulated. Sox2 binding sites in ES cells, for example, also form 3D clusters in nuclear regions that are segregated from heterochromatin but enriched in RNAP II. Such heterogeneous distribution impacts Sox2’s target search strategy by reducing the global search efficiency while allowing for flexible local adjustments [65]. Along the same line, P-TEFb has been found to sample the nuclear space during its target search in a position-dependent fashion, constrained by its interactions with the hierarchical structures in the crowded nuclear environment; c-Myc, on the other hand, explores the nuclear space more freely and has an equal probability of reaching any target regardless of its location (Fig. 3b) [69]. The abundance of patterns such as these suggests that spatial localization dynamics, whether evanescent or long-lived, constitute a general regulatory mechanism of gene expression via higher-order nuclear architecture.

Another mode of spatial organization of TF activity takes place in the form of nucleo-cytoplasmic translocation, as exemplified by NF-κB, a key nuclear regulator of cellular stress responses. Upon activation, NF-κB exhibits periodic oscillations of nuclear import from the cytoplasm (Fig. 3c), which in turn controls the transcription of IκB-α via a negative feedback loop [100]. Such oscillations resonate in some way with the transcriptional pulses aforementioned, although they differ markedly in their timescales, synchronicity, periodicity (or lack thereof), and mode of operation. Like transcriptional pulsing, many of the parameters of NF-κB translocation, including amplitude, response time, and number of oscillations, can be regulated. Among the variety of regulatory factors are both the frequency and strength of the stimulation signal [101–103] as well as mechanical features such as cell shape and microenvironment [104]. Importantly, changing the translocation dynamics could impact the expression profiles of NF-κB target genes in both cultured cells as well as live animals [101, 102, 105], pointing to functional roles of such oscillatory nuclear translocation.

Similar translocation phenomena have been observed for other TFs as well. Oct4, for example, undergoes facilitated nucleo-cytoplasmic transport in early developing embryos. However, fluorescence decay after photoactivation (FDAP) measurements revealed two sub-populations of cells within the embryo that exhibit distinct rates of nuclear export and import as well as an immobile fraction of Oct4; cells with slower Oct4 kinetics are more likely to give rise to the pluripotent cell lineage later on, while those with faster kinetics contribute mostly to the extra-embryonic lineage [80]. For the transcription factor NFAT, nuclear translocation is manifested in two different ways for its two isoforms: the NFAT1 translocation pulses are synchronized and amplitude-modulated, whereas those of NFAT4 are unsynchronized and frequency-modulated [106]. Such complementary strategies for spatial translocation dynamics allow the TF isoforms to broaden the modality range of mammalian cells to respond to external signals.

Spatial organization is also not restricted only to the protein molecules involved in transcription; the structure and positioning of chromatin DNA plays an equally important role in transcriptional regulation [107, 108]. In addition to packing the genome at the level of euchromatin/heterochromatin and thus modulating the accessibility of the DNA to regulatory proteins and RNA polymerases, chromatin structure is dynamically organized at multiple levels within the three-dimensional nuclear space. For example, long-range contacts between the promoter and enhancer of the β-globin gene established through chromatin looping have been shown to impact the bursting kinetics of its transcription [81]. On a grander scale, interphase chromosomes are known to occupy distinct regions of the nucleus termed “chromosome territories” [109]. Such compartmentalization imposes spatial constraints on where transcription occurs, as actively transcribed genes tend to be localized near the periphery of these territories, whereas noncoding regions are either stochastically distributed or preferentially localized near the interior of the territories [110–112]. On the other hand, the positioning of genes to the nuclear periphery, where the chromatin adopts a more compact structure, leads to repression of their transcription as a consequence of nuclear lamina-mediated silencing [113]. More recently, it has been shown that the entire X chromosome could be recruited to the nuclear lamina by the long noncoding RNA Xist to achieve chromosome-wide transcriptional silencing [114]. These examples collectively demonstrate the diverse array of regulatory modes enabled by the hierarchical organization of mammalian genomes in the nuclear space.

## Regulating transcriptional dynamics through concentration, oligomerization, or epigenetic modifications

**Fig. 4.**
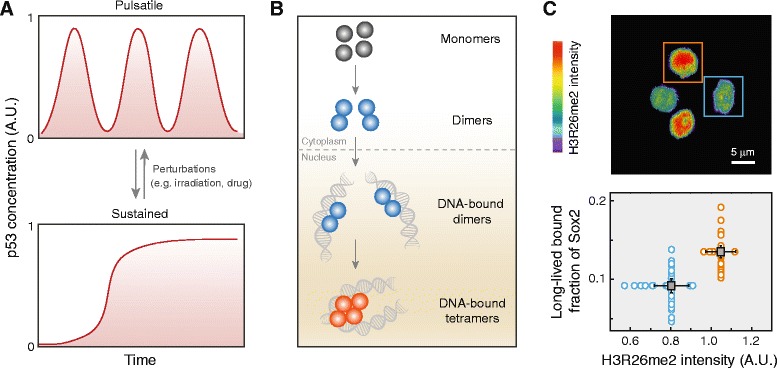
Organizing mammalian transcriptional dynamics through modulating concentration, oligomerization, or epigenetic states. **a** Intranuclear concentration of p53 can exhibit either pulsatile (*top*) or sustained (*bottom*) changes when perturbed with different stimuli; cells with pulsatile response recover from DNA damage whereas those with sustained response enter senescence. **b** Regulating TF mobility and function through the dynamic interconversion among its monomeric, dimeric, and tetrameric forms. Depicted here is the case of STAT3, whose dimers must first translocate to the nucleus and bind DNA before forming tetramers, which could then amplify or repress STAT3 dimer-mediated transcription. **c** Immunofluorescence staining of four-cell mouse embryos reveals distinct differences in H3R26me2 levels (*top*); cells with a higher level (*orange*) exhibit a larger fraction of Sox2 engaged in long-lived DNA-binding than those with a lower level (*blue*) (*bottom*). Adapted from [118] (**a**), [124] (**b**), and [66] (**c**) with modifications

In addition to being organized in the temporal and spatial realms, transcriptional dynamics can also be regulated through changes in the concentration of TFs. The tumor suppressor p53, for instance, responds to stress such as DNA damage with oscillatory pulses in its concentration [115, 116]. Basal pulses of p53 in proliferating human cells are responsible for attaining the balance between high sensitivity to sustained stress and tolerance to transient DNA damage during normal growth [117]. Moreover, the temporal profile of such concentration changes can be modulated with different external stimuli (e.g. UV versus γ-radiation) (Fig. 4a), which subsequently alters the expression of p53 target genes and could lead to different cell fates [118, 119]. In noisy environments with large cell-to-cell concentration variability, mammalian systems have also evolved strategies to sense and regulate not absolute concentrations but fold changes in concentration, such as in the case of NF-κB [120], thereby allowing cells to buffer against stochastic concentration fluctuations.

The oligomerization state of a TF is another factor that may impact its mobility and function. For example, FCS measurements of p53 in single cells reveal that the TF exists in a mixture of oligomeric states; their rapid homo-tetramerization upon DNA damage activates the transcriptional targets of p53 [121]. Similar strategies have also been observed for the activation of the Rac1 signaling pathway [122], interaction between HP1-α and heterochromatin [123], and DNA-binding of STAT3 upon nuclear translocation [124] (Fig. 4b). These diverse cases not only establish the modulation of oligomerization states as an effective mechanism for regulating TF function, but also demonstrate the unique prowess of fluctuation-based imaging techniques (such as FCS and N&B analysis), which remain as the only methods suitable for probing this aspect of nuclear dynamics.

Lastly, the interplay between transcriptional dynamics and epigenetic modifications are also beginning to be probed at the quantitative level. Recently, it has been found in live cells that histone H3 lysine-27 acetylation could both facilitate the target search of transcriptional activators and promote the transition of RNAP II from the initiation form to the elongation form [125]. H3 arginine-26 methylation, on the other hand, regulates the long-lived DNA-binding of Sox2 in developing embryos by controlling chromatin accessibility (Fig. 4c) [66]. Moreover, by monitoring the dynamics of repressive chromatin regulators associated with various DNA and histone modifications in single cells, epigenetic silencing and reactivation events were found to occur in a stochastic fashion. These regulators exhibit distinct timescales of epigenetic memory, and regulation is achieved by changing the fraction of cells silenced rather than the level of transcription [126].

## Conclusion and outlook

We are now at the exciting threshold where advances in quantitative single-cell imaging have empowered us with the means to explore the spatio-temporal dynamics of transcription in unprecedented detail. By generating spatial maps and temporal trajectories at high resolutions in diverse living systems, these approaches not only furnish or revise previous biochemical models with physical parameters and insights, but also shed new light on the multi-faceted landscape for organizing transcription that is shared by mammalian systems ranging from single cells to developing embryos.

The transcription-related processes reviewed here constitute only a small fraction of the functions carried out in the mammalian cell nucleus; other equally important and complex processes, such as RNA processing and export, chromatin organization and remodeling, and genome replication and maintenance, are all heavily regulated in both time and space through mechanisms just as versatile as those outlined above. To probe these processes both on their own and in cooperation with each other requires the continuous pushing of the resolution and sensitivity limits of imaging techniques, combined with novel approaches to monitor a large number of molecular species in the nucleus simultaneously. To that end, the recently developed methods capable of highly multiplexed imaging of either protein, DNA, or RNA species offer exciting hopes [127–129]. The ability to image chromatin DNA in vivo in a label-free manner [130, 131] also points out new directions that obviate the necessity of using bulky and often perturbative fluorescent proteins or other organic probes. The more important thing, however, is to go beyond the cultured cell systems and apply these emerging techniques to more physiologically relevant contexts, such as developing embryos, tissue explants, or even live animals, so as to generate newer insights into mammalian nuclear dynamics in vivo for many more years to come.
